# Moments of deep connection between clinicians and patients in oncology: a mixed-methods study of sacred moments

**DOI:** 10.3389/fpsyg.2026.1833697

**Published:** 2026-06-09

**Authors:** Faiza Yasin, Leah Puklin, Martha Quinn, Kathryn Norman, Sanjay Saint, Tara Sanft

**Affiliations:** 1 Department of Medicine (Medical Oncology), Yale School of Medicine, New Haven, CT, United States; 2 Yale School of Public Health, New Haven, CT, United States; 3 School of Public Health, University of Michigan, Ann Arbor, MI, United States; 4 Veterans Affairs (VA) Ann Arbor Healthcare System, Ann Arbor, MI, United States; 5 Department of Internal Medicine, University of Michigan Medical School, Ann Arbor, MI, United States

**Keywords:** career fulfillment, meaning, oncology, patient provider communication, sacred moments

## Abstract

**Purpose:**

Sacred moments are periods of deep connection between clinicians and patients, associated with increased clinician career fulfillment and stronger therapeutic alliances in radiation oncology and internal medicine. Our study is the first to investigate sacred moments in medical oncology.

**Methods:**

We conducted semi-structured qualitative interviews with clinicians and patients at an NCI-designated Comprehensive Cancer Center. Interviews were recorded, transcribed, and analyzed using inductive coding. Participants completed a closed-ended (Likert-style) questionnaire that assessed demographics, and the frequency and impact of sacred moments.

**Results:**

Participants included 24 clinicians and 10 patients. All participants reported having experienced a sacred moment. All clinicians agreed that when they experienced a sacred moment, they felt professionally fulfilled (100%), connected to their patient (100%), and were reminded why they went into oncology (100%). Patients felt connected to their oncologist (88%), had trust in (100%) and felt cared for (100%) by their oncology team.

Three primary themes emerged from qualitative interviews: (1) Sacred moment catalysts, (2) Impacts, and (3) Cultivation strategies. Key catalysts included pivotal clinical junctures (such as initial cancer diagnosis and end of life), clinician preparedness and expertise, and longitudinal care relationships. Sacred moments enhanced career fulfillment among clinicians, promoted goal-aligned clinical care decisions, and strengthened patients' sense of being cared for as a whole person. Cultivation strategies centered on efforts to learn about patients beyond their cancer diagnosis, values-based clinical training and mentorship, and workplace culture.

**Conclusion:**

Experiencing sacred moments provides oncology clinicians with professional fulfillment and reinvigorates engagement. For patients, these moments build trust and foster goal-aligned care.

## Introduction

The current U.S. healthcare landscape can be fragmented and dissatisfying from both the patient and clinician perspective (Kern et al., 2019; Hero et al., 2016; Noseworthy, 2019). This disconnect is particularly impactful in oncology, where meaningful clinician-patient relationships are essential for navigating complex treatment decisions. Moral injury and burnout are high among oncologists ranging from 32% to 73% (Martinez-Calderon et al., 2024; HaGani et al., 2022; Singh et al., 2022; Medisauskaite and Kamau, 2017) and rising over time (Schenkel et al., 2025), threatening the sustainability of cancer care. As the current healthcare environment includes increasing bureaucratic burdens (Noseworthy, 2019), innumerable healthcare metrics, and a projected shortage of oncologists (Yang et al., 2014), interventions to enhance professional fulfillment and care satisfaction are needed (Murali and Banerjee, 2018). One promising avenue is in understanding and cultivating meaningful authentic clinician-patient relationships.

Sacred moments are characterized by deep connection or profoundly meaningful exchanges between clinicians and patients, and are integral to high-quality cancer care (Gilligan et al., 2017; Collet et al., 2022). They refer to discrete, temporally bounded instances of deep interconnectedness (Lomax et al., 2011), boundlessness where “time stood still,” transcendence or being “set apart” from others, spiritual emotion, and ultimacy (Quinn et al., 2023; Radetsky, 1985; Pargament et al., 2014). Prior studies have found defining themes to include a sense of profound meaning (Quinn et al., 2023), emotional resonance (Quinn et al., 2023), and deep feelings of shared humanity (Blödt et al., 2021). They often occur during critical illness or around end of life, and during unstructured clinical encounters (Quinn et al., 2023). The cross-cultural replication of sacred moments in non-US based settings supports the construct's validity and universality (Sakama et al., 2026). These elements together distinguish sacred moments from routine clinical interactions, and from related adjacent constructs such as general empathy or therapeutic rapport. Both patients and clinicians experience these moments (Ameling et al., 2025), which have been associated with better psychosocial outcomes (Jewett et al., 2022), enhanced wellbeing (Ameling et al., 2025; Goldstein, 2007), reduced distress (Riba et al., 2019; Phelps et al., 2012), and strengthened therapeutic relationships (Quinn et al., 2023; Kienle et al., 2018). For clinicians, they may protect against moral injury and increase a sense of meaning in their work (Quinn et al., 2023; Ameling et al., 2025).

While sacred moments have been evaluated in internal medicine (Quinn et al., 2023; Ameling et al., 2025) and radiation oncology (Saint et al., 2024), they have not been explored in medical oncology, where relationship-centered care is crucial and longitudinal. This study is the first to use both quantitative and qualitative methods to describe the frequency and nature of sacred moments and elucidate themes related to experiencing these moments among patients and clinicians in oncology.

## Methods

### Ethics approval

Approval was obtained from the Yale Institutional Review Board (Protocol No. 2000037190).

### Clinician cohort

We used purposeful sampling to identify oncology clinicians at a single National Cancer Institute Designated Cancer Center, Smilow Cancer Center at Yale New Haven Hospital. These included attending physicians, fellows, and advanced practice providers (APPs). We included clinicians with subspecialties in Medical Oncology, Hematology, Surgical Oncology, and Hospice and Palliative Medicine. Hematology and Medical Oncology are combined into “Oncology” throughout the remainder of this manuscript for simplicity. All clinicians in the aforementioned departments were invited to participate by email invitation by the author (F. Y.) between March 2024 and February 2025 with additional in-person recruitment through cancer center faculty meetings and fellowship conferences by two investigators (F. Y., T. S.).

### Patient cohort

Patients were identified in the outpatient oncology clinic setting by convenience sampling and invited to participate by one investigator (F. Y.) at an in-person clinical encounter. The treating oncologist gave permission to approach eligible patients. Interested patients were contacted by phone to schedule a video interview at their convenience.

### Study design overview

This study used a qualitative-dominant mixed-methods design with a convergent approach, in which semi-structured qualitative interviews were the primary method of inquiry, and a brief closed-ended questionnaire provided descriptive context regarding the frequency and perceived impact of participants' reported experiences. The quantitative component was intended to complement rather than triangulate qualitative findings. Integration occurred at the interpretation stage, where quantitative results were used to frame the qualitative themes.

### Qualitative: data collection and analysis

Interviews with clinicians and patients were conducted between March 2024 and February 2025 by the author (F. Y.). All interviews were conducted via Zoom video and were audio recorded. Informed consent was obtained verbally for all participants at the start of each interview. We continued to sample participants until a saturation of themes was attained, defined as the point at which no new codes or themes were emerging across successive transcripts. Saturation was assessed iteratively during coding by the investigator team (F. Y., L. P., and T. S.) and were considered separately for the clinician and patient cohorts. All interviews were transcribed verbatim and de-identified. Clinicians and patients were interviewed using a semi-structured interview guide adapted from Quinn et al. (Supplementary Table 1; Quinn et al., 2023). The interview guide informed data collection only, while the textual data was analyzed using an independent inductive thematic analysis (Braun and Clarke, 2014) approach (Quinn et al., 2023; Saint et al., 2024; Coates et al., 2021). An inductive approach was selected given the exploratory nature of the study, and the absence of prior work on sacred moments in medical oncology, allowing themes to emerge directly from participant accounts rather than from a predetermined framework. Two authors (F. Y., L. P.) independently read and analyzed one transcript each to begin codebook development. The investigator team (F. Y., L. P., T. S.) discussed the coding strategy and refined the codebook in an iterative process. Differences in applied coding were discussed and agreement was reached by consensus for an aligned approach. The process was repeated with three additional transcripts. Emerging themes were evaluated concurrently and newly added codes were applied to the previous transcripts by the investigator team. The remaining transcripts were then divided between two authors (F. Y., L. P.) for coding. After all transcripts were coded, a third investigator (T. S.) conducted a secondary review on five randomly selected transcripts. Discrepancies in coding were discussed by the investigator team and resolved through mutual agreement. Findings were organized into primary themes and sub-themes derived from the developed codebook. Dedoose (Version10.0.25) software was used for secure data management, storage, and data analysis.

### Quantitative: data collected and analysis

Participants were emailed a brief questionnaire with responses measured on a five-point Likert scale (Supplementary Tables 2, 3). Questionnaires included demographic data and asked about the frequency of experiencing sacred moments. Participant characteristics were summarized using descriptive statistics (means and frequencies). Clinician survey data and patient survey data were analyzed independently, using similar descriptive statistics.

### Reflexivity

Throughout the study, the research team engaged in reflexivity by critically examining how our individual backgrounds and assumptions may have influenced the data collection, interpretation, and representation of findings. These reflexive practices were intended to enhance transparency, credibility, and rigor in the qualitative analysis.

The lead investigators (F. Y. and T. S.) are practicing clinical medical oncologists, and one investigator (F. Y) conducted all interviews when she was a fellowship trainee. This positionality may have influenced participant recruitment, as patients were approached during clinical encounters, and may have shaped interview dynamics, the direction of conversation, and predominantly positive framing of sacred moments. To mitigate these influences, independent coding was performed by a second analyst (L. P.) who is a doctoral-level researcher with expertise in implementation science and qualitative research in medical oncology. An iterative, consensus based approach to theme development was utilized throughout the analytic process. These potential influences are also acknowledged in the Limitations Section.

## Results

### Participant characteristics

A total of 24 clinicians were interviewed; 23 completed the questionnaire. Mean age was 46.1 years (range 32–68, *SD* = 11.5). Clinician experience ranged from current trainees (*n* = 5, 20.8%) to >15 years (*n* = 7, 29.2%) of post-training years in practice. A total of 10 patients were interviewed; eight completed the questionnaire. The mean age was 68.3 years (range 53–81, *SD* = 8.8). Most patients had solid tumor diagnoses (*n* = 9, 90.0%), and half (*n* = 5, 50%) had metastatic disease (Table 1 for all participant characteristics).

**Table 1:** Study participant characteristics.

Clinician cohort (*n* = 24)	
Mean age, years (range)	46.1 years (range: 32–68), *SD* = 11.5
Sex (*n*, %)	
Male	8 (33.3%)
Female	16 (66.7%)
Clinician type (*n*, %)	
Physician	22 (91.7%)
Advanced Practice Provider (APP)	2 (8.3%)
Years in practice (*n*, %)	
Trainee	5 (20.8%)
0–5 years	5 (20.8%)
6–10 years	4 (16.7%)
11–15 years	3 (12.5%)
>15 years	7 (29.2%)
Specialty (*n*, %)	
Oncology	17 (70.8%)
Surgical oncology	3 (12.5%)
Hospice and palliative medicine	4 (16.7%)
Patient cohort (*n* = 10)	
Mean age, years (range)	68.3 years (range: 53–81), *SD* = 8.8
Sex (*n*, %)	
Male	6 (60%)
Female	4 (40%)
Cancer stage (*n*, %)	
Metastatic	4 (40%)
Non-metastatic	5 (50%)
Not applicable	1 (10%)
Cancer type *(n*, %)	
Solid tumor (Oncology)	9 (90.0%)
Ovarian cancer, breast cancer, lung adenocarcinoma, colon adenocarcinoma, and cholangiocarcinoma	
Malignant hematology	1 (10.0%)
Multiple Myeloma	

### Quantitative findings

All clinicians had experienced a sacred moment, and most experienced them at least monthly (*n* = 22/23, 95.6%). All clinicians agreed that when they experienced a sacred moment they felt professionally fulfilled (*n* = 23/23, 100%), connected to their patient (*n* = 23/23, 100%), and this allowed them to remember why they went into cancer care (*n* = 23/23, 100%). Most felt they were always or often (*n* = 19/23, 82.6%) able to practice in alignment with their values, and that their work was always or often (*n* = 23/23, 100%) meaningful (Table 2).

**Table 2:** Clinician survey data.

Clinician cohort (*n* = 24 with 23 respondents)							
Frequency of sacred moments							
1. (Never)	0 (0.0%)						
2. (Rarely, yearly)	1 (4.3%)						
3. (Sometimes, monthly)	12 (52.2%)						
4. (Often, weekly)	9 (39.1%)						
5. (Always, once or twice per clinic)	1 (4.3%)						
	Always	Often	Sometimes	Rarely	Never	Mean	SD
**Values**	7 (30%)	12 (52%)	4 (17%)	0 (0.0%)	0 (0.0%)	4.13	0.68
I am able to practice medicine in a way that reflects my values.							
**Meaning**	10 (43%)	13 (57%)	0 (0.0%)	0 (0.0%)	0 (0.0%)	4.43	0.50
My work is meaningful to me.							
	Strongly agree	Agree	Neutral	Disagree	Strongly disagree	Mean	SD
**Fulfillment**	19 (83%)	4 (17%)	0 (0.0%)	0 (0.0%)	0 (0.0%)	4.83	0.38
When I experience a sacred moment, I feel professionally fulfilled.							
**Connection**	19 (83%)	4 (17%)	0 (0.0%)	0 (0.0%)	0 (0.0%)	4.83	0.38
When I experience a sacred moment, I feel connected to my patient.							
**Why**	16 (70%)	7 (30%)	0 (0.0%)	0 (0.0%)	0 (0.0%)	4.70	0.46
When I experience a sacred moment, I remember why I went into oncology.							

All patients experienced sacred moments at least sometimes (*n* = 8/8, 100%). Most agreed that when they experienced a sacred moment they felt connected to their oncology provider (*n* = 7/8, 88%), had trust in their team (*n* = 8/8, 100%), and felt cared for by their team (*n* = 8/8, 100%; Table 3).

**Table 3:** Patient survey data.

Patient cohort (*N* = 10 with eight respondents)							
Frequency of sacred moments							
**1 (Never)**	0 (0.0%)						
**2 (Rarely)**	0 (0.0%)						
**3 (Sometimes)**	5 (62.5%)						
**4 (Often)**	2 (25.0%)						
**5 (Always)**	1 (12.5%)						
	Strongly agree	Agree	Neutral	Disagree	Strongly disagree	Mean	*SD*
**Connection**	4 (50%)	3 (38%)	1 (13%)	0 (0%)	0 (0%)	4.38	0.70
When I experience a sacred moment, I feel connected to my oncology provider							
**Trust**	7 (88%)	1 (13%)	0 (0%)	0 (0%)	0 (0%)	4.88	0.33
I have trust in my oncology care team							
**Cared**	6 (75%)	2 (25%)	0 (0%)	0 (0%)	0 (0%)	4.75	0.43
I feel cared for by my oncology care team							

### Qualitative findings

We found that sacred moments were commonly experienced by both clinicians and patients, with the emergence of three primary themes: (1) sacred moment catalysts, or common conditions and situations when sacred moments occur, (2) impacts of sacred moments, or how these moments affected the participant(s), and (3) strategies for cultivating sacred moments (Tables 4, 5 for detailed thematic results and illustrative quotations).

**Table 4:** Sacred moments domains and themes.

Catalysts	Impacts	Cultivation strategies
• **Pivotal point in clinical care**—these include initial cancer diagnosis, disease progression, end of life, clinical decline, and good outcome • **Preparedness and expertise**—ability to convey preparedness for a clinical visit or clinical expertise • **Organic**—spontaneous moments that unfold naturally • **Being present**—investment of self, intention • **Shared or similar characteristics with a patient or clinician**—this may include shared experiences, age, and hobbies • **Continuity**—duration of follow up, building relationship over time, and familiarity • **Memorable moment that inspired an intense emotion**—laughter, tears, joy, and gratitude	**PATIENTS** • **Sense of being cared for**—gratitude for care, feeling heard in their care and goals being valued newline **CLINICIANS** • **Career fulfillment**—career meaning and purpose, experiences that keeps one going in their field newline **SHARED** • **Goal aligned care**—clinical care that is aligned with patient goals of care and values • **Trust building**—building rapport and trust between a clinician and patient	• **Culture, training, and mentorship**—physician residency and fellowship training, senior role models, cultural values of the institution one practices in • **Building a relationship**—efforts to create a patient-clinician relationship • **Knowing someone beyond their diagnosis**—recognizing shared humanity, and understanding what gives a patient meaning or quality of life • **Partnership**—clinicians meeting patients where they are at, alignment of goals

**Table 5:** Sacred moments illustrative quotes.

Sacred moment catalysts	
Pivotal point in clinical care	“*It was a bit of a roller coaster. And I feel like that lends itself to patients or their families being understandably concerned and upset and unsure. And having cancer in general will obviously prompt that in many people, but more so if there are these fluctuations, and you're doing better, you're doing worse, you're doing better. And so I feel like that kind of lends itself to, you know, clinicians potentially playing a more important role*” (Clinician FD81F3A0).
	“*When I diagnosed her, it felt like it was like the end of the world. And then I saw her for her pre-op visit. She had neoadjuvant chemotherapy… And her tumor had shrunk. Like had disappeared! Like she had had a giant tumor. And it disappeared. I went back to my note to make sure I knew what side her tumor had been on and went back to her, and it was gone. And just like in her eyes and the way that I felt of like, my God, like it might be gone…*” (Clinician EEE8F431).
	“*I was taking care of a lady in her 70s, and her husband was very involved in her care. She was… participating in our clinical trial, and was in complete hematologic remission. And suddenly this patient deteriorated. I directly admitted her to the hospital. And the patient… succumbed to this unrelated complication, which was unrelated to her hematologic diagnosis. And, we were in the early to mid-COVID period, where we had all the restrictions. And, it's particularly, troubling, when you lose a patient who is in remission. We had done everything to achieve that state. So it was just very painful, for obviously the family, and this husband who, we stood in the room in the ICU… I think we were both crying. And I hugged him despite all the precautions and the masking, and all of that. And we kind of expressed our emotions by standing there and sobbing, as she was going downhill and there was no return*” (Clinician 1CB0F1D2).
	“*And then again, to take the time to really explain the whole process and the whole disease and then, you know, lay out the future of it all in that same meeting where you could then understand it*” (Patient 772626DF).
Preparedness, expertise	“*I think that is also something that instills a lot of trust in people when you come in to a visit educated. All that together lays the groundwork for some degree of trust that the patient already has in you… just based on your level of preparedness for the visit…*” (Clinician C15EB1A4).
	“*It always helps when somebody knows… like your background and knows your latest numbers and everything, when they come to you with that, it's like, all right, you're on my side and you know my history and you're just sticking with me*” (Patient AF113363).
	“…* because he was very confident, and he said, we're going to zap it and get rid of it. He was just so confident. We left the office feeling like, no problem*” (Patient 17C4F9F0).
	“*I had a lot of moments of connectedness with him because he was so thorough. He was really, really thorough. And I think to his own people working with him sometimes, I think to his detriment, like he's a pain in the ass, but it just felt like you were in good hands, because he was so damn thorough with everything*” (Patient AF113363).
	“*For some reason, I was really comforted by it. Like, this guy's got it. He's got the wheel. I'm just going to let go. I just had the feeling from how thorough he was that he was just the best. That I was getting the best*” (Patient AF113363).
	“*I got brought up from [outside hospital] at like 12:30 at night. He met me in my room and reviewed the whole process for over an hour. My wife called at 1:30 a.m. because she found out I was moved, and he started the whole thing over for another hour. And she was like, that guy's thorough. And I was like, yeah. And that was just the way it was the whole way through, you know? It was just like…then you can throw your arms around the shoulders and say, all right, carry me along. You know, because I know you've got all your bases covered*” (Patient AF113363).
Organic	“*it's a tough thing to try to encourage to happen. I feel like it happens… extremely organically. If both parties are open to it, it may well-happen. I don't know if it's something you actively seek out or it just kind of happens when it happens. I feel like it's so organic most of the time that, that connection between a patient and a provider, it's either going to happen or not. And again, this is very like many other interpersonal interactions or relationships*” (Clinician FD81F3A0).
	“*You know, human relationships are fascinating. Because there's some level where we can relate to some people more than others, right?*” (Clinician 640F317D).
	“*but the other thing is there's something, when you walk into a room and before you even talk to someone, there's a connection, right?*” (Clinician A1EDB3C6).
Being present	“*I had at one point I had asked her kids names. I think there were five or six kids. And then I had been like, I should memorize the kids names, so I did. So the next time I had seen her, I asked her about the kids using their names. I had like done that to show her that I was like thinking about them*” (Clinician A75B688A).
	“*Whenever we meet every 2 weeks, it's my time. I never feel like the doctor is rushed. If we need to speak to him for an hour, we know he'd be there for an hour. I never had that rushed feeling that I have some other specialties where it's just… I gave you my 30 s, next, but he will take the time. And never, ever, ever have I ever felt rushed*” (Patient B8E0EF66).
Shared or similar characteristics with a patient or clinician	“*And I just heard the fear in his voice, and maybe because the patient was the same, maybe a few years older than my dad. And so I remember talking to the ED and asking can we compromise? And if it wasn't for moments of [connection] that I had had already like that, I don't know that I would have felt like, empowered to you know, make that decision*” (Clinician 6369EB71).
	“*She's a nurse, an ED nurse. And like I said, she is 38 and so am I*” (Clinician 510C0518).
	“*It gives you a better connection with the doctor, realizing that, he certainly has the same type of thing going on in his life that we have going on in ours*” (Patient 0862035D).
	“*I could personally relate to, in that I was an athlete and on the track team when I was in high school also. And so maybe there was some part of me that sort of understood the meaning or value behind what he was saying*” (Clinician F22AE36E).
	“*I do think that it was because she was a young… I think she was my age and she was a young woman. There was a relational thing like that*” (Clinician A75B688A).
	“*But this is over the span of many years, and we followed [her]. I remember, you know, when we would walk into the room, she would be knitting something, and I talked to her about my mother-in-law who knits all the time, and we talked about those things…*” (Clinician 2CB438F0).
Continuity	“*[P]art of it's the longevity of the relationship. The fact that I had many interactions with her over the course of months and longer. And not just the frequency or longevity, but also the, the course of her illness*” (Clinician FD81F3A0).
	“*I'm not sure why this stands out but, I've known him for a few years, so it's seeing him month after month for years…you start to know people*” (Clinician F22AE36E).
Memorable moment inspiring intense emotion	“ *The patient had had an MRI and his wife always teased him. He had like a brain MRI. I remember she said like, how did it look? And everything was okay. And then I can't remember exactly how it was worded, but somehow I made a joke like that there was that, you know, the brain wasn't there or something. And she just cracked up. She loved it. And he loved it too. Cause they were like so happy that that like, they already knew the news was good… but there are like these times when you can like make a joke that kind of also is a transcendental…*” (Clinician A75B688A).
	“*And he just gives you this real warm, happy, secure feeling, you know, not all doctors give you that secure feeling. And that in itself is really big*” (Patient C4AF1D28).
	“*And I told [my wife], I can't wait for chemo. And she goes to Dr. [Oncologist Name] the next day*, “*Doctor, have you ever had anybody tell you they're really looking forward to chemo?*” *And he goes*, “*No*.” *And she just kind of pointed to me. [laughter] You know, it was just kind of a funny moment*” (Patient B8E0EF66).
**Impacts of sacred moments**	
**PATIENT IMPACTS**	
Sense of being cared for	“*You feel like the doctor or nurse care team has your best interest at heart. So if they can address this part of you, you have total confidence in their ability to give you the best medical advice as well. If they're paying attention to all these other details, you're inspired and confident that they're paying attention to the medical details too…*” (Patient B8E0EF66).
	“*I think if you have a clinician that's more empathic, and connected, and present, you're going to feel more seen and more heard and more understood, whether there's good or bad news to be shared*” (Patient 67018C96).
	“…*for the patient, that you're actually in a room with someone who cares. That's basically it*” (Patient D5146DFC).
	“*I have felt that [my husband] is a human being first and a patient second. He is not cancer. He's a person that has a whole life and a whole story. And cancer is just one small part of that*” (Patient B8E0EF66).
**CLINICIAN IMPACTS**	
Career fulfillment	“…*even though parts of it can be difficult, it's also rejuvenating, right? So it drives, it reinvigorates passion for our jobs and reminds us that we have skills and things to bring to our environment*” (Clinician EEE8F431).
	“*I think it gives added meaning and importance to the work that I do. I know that I'm there at really difficult and important times in people's lives. And I value that*” (Clinician 67018C96).
	“*This is why we do this. There are a lot of things that are distractors and frustrations, ranging from our schedules, the volume of work we do, the electronic medical record, all of the fragmentation and disruption in the system, dealing with peer reviews for coverage for scans and tests and procedures and you know, the list goes on, but the ability to connect in a really powerful and meaningful way with an individual and families is deeply rewarding and in surgery particularly, but also in medical oncology, when you have the sense that the power of your expertise and the trust you have in your own training is going to deliver measurable benefit to this individual is a sense of deep satisfaction*” (Clinician C96443BD).
	“*I would say this is one of the, if not the most important reason, that I do what I do. If I really didn't deeply connect with my patients, or have these kind of moments, it would be a really hard job. It would be a very unfulfilling job. All my patients die. All my patients die as a palliative care clinician*” (Clinician 510C0518).
	“*I think these are the moments that can carry me forward when I'm experiencing other less pleasant moments, there's certainly plenty of those too. I feel like these are the kinds of things which can at least mitigate some of the burnout that we can experience in the course of our days and careers*” (Clinician 7D22F31F).
	“*I think it gives us more energy and motivation to then tackle the next thing…like these little moments of feeling close or bonding, then you want to go to bat for that person. You feel like you know them, they're not just a faceless person in your clinic, which nobody really is, but I do think taking the time to notice these moments or really be present for them helps see each person as a human and then allows you to do the hard work, which is usually behind the scenes, in the workroom, on the phone, in the in-baskets, fighting for what you think that person needs*” (Clinician ABDC1A68).
	“*I was just thinking like here's this patient who could have been my grandad, and he's going to be able to go home and pass away the way he'd want to, with his family. And knowing that I was able to help play a role in that was really huge. And I remember thinking… you hold on to moments like that when nothing else kind of seems to go right*” (Clinician 6369EB71).
	“…*our patients give us so much strength and energy, and there's no question that those relationships really keep us on top of the water and not sinking. Or if we're sinking, they can be a life breath to bring us back up. It's such a fascinating sort of symbiosis really*” (Clinician 640F317D).
**SHARED IMPACTS**	
Goal aligned care, complex decision making	“*if you're having some other shared connection, then maybe there is that sense of this person is responsible for me and, I will do everything I can to make sure that we still have the shared goal*” (Clinician D5146DFC).
	“*It probably impacts things like shared decision making a little bit more. When you get to know somebody on a deeper level, I might have a feeling of certain treatment approaches that maybe would or would not jive with them*” (Clinician F22AE36E).
	“*When you know who that person is as a person, and what matters most to them, I think you act differently, or you change behavior, or your decision making. I think it probably plays a large role into the actual decisions that are being made, and as such, the outcomes that matter to the patient*” (Clinician 510C0518).
Trust building	“*I anticipate, or I would imagine that patients are more likely to take your word for things, like understand if say you don't call them the day after scan that you're going to get to that, you're going to call them at some point. So there's some level of, this has to do probably with trust, … connectedness or… just trust that you're going to, your intentions are going to be good going forward*” (Clinician C15EB1A4).
	“*I think they are trust building ultimately… I think if you can have that type of a deep moment of connection with someone, however that is, I think you're more likely to trust them. So that when the next juncture comes where you have to make a recommendation, whether it be for a new therapy, or maybe it would be that more therapy is more likely to be harmful. My hypothesis, and I will probably pay attention more to it, but I think when those connections are there… you can kind of compare, like how when you're doing a consult in the hospital and you walk in, and you're trying to have a hospice discussion with a patient you don't know… how different that feels compared to like, when you have that relationship. I think it's, it's different*” (Clinician 26FBEB18).
	“*to have the sense that someone cares for you as an individual, not just as a clinical problem and listen to you and appreciates your humanity, I think gives people an enormous sense of relief and comfort and security. And again, trust*” (Clinician C96443BD).
	“*I found that when have those moments of connection, and that trust is built, it's kind of everything*” (Clinician 6369EB71).
**Cultivation strategies**	
Culture, training, mentorship	“*It's modeling behavior. It's to say to the med student, that was really hard for me, because this young person reminds me of my friend, who also had cancer, or the person is alone and has no support system. So I think modeling it in that way, both informally as you're teaching trainees, but also in a slightly more organized way through advanced training. But also allowing for nuance, not a module. That shows that this is beneficial, that it's impactful, that it allows both the physician and the patient, or the provider and the patient to form a bond…*” (Clinician EEE8F431).
	“*I do think that training, in some ways is an important part of it, and specifically, having the people who train you also value these sorts of moments, but also just value the patient as the human being first in all of their care. I think I have been very lucky in the people who I've learned from, and trained with, who have really made me focus first on the fact that the person who is sitting in front of me is a person with a life, and a family, and an entire wealth of experiences and knowledge and wisdom to share, and what I am there to treat in terms of their physical element is one part of the whole human being, and I think because that has been emphasized to me so much throughout my training, that it's essentially just a part of how I provide care in general, as an automatic, as second nature*” (Clinician 192DE0E2).
	“*But it is good to look at this because maybe some of these things that are able to be sort of codified or defined better, can be taught. We're all capable. So maybe that's the other way I would add that maybe some of the insights you garner. Or can have them be distilled to be a little bit more objective. Maybe that's something that we can teach*” (Clinician 640F317D).
Building a relationship, time spent	“*These people were busy. But they always, the good ones, the people you feel connected to, the ones you feel most confident about your care, are the ones that take the time*” (Patient B8E0EF66).
	“*I think the best care providers look for ways to make connections. And it's a two way street. It's not just like us talking about us. Like he talks about himself. He talks about his family. Like I said, I keep track of how many iPads his kids have lost on transcontinental flights*” (Patient B8E0EF66).
	“*I think what creates relationships is one-on-one time, and your ability to speak with the patient, and spend time, and talk, not only about the numbers or the chemo, or the blood counts, but also share some human interaction. Learn about their interests, their goals, their family…*” (Clinician 1CB0F1D2).
	“*I think there is that chance to grow the relationship over time with those patients. And it can be less of a big transcendental moment…more of the accumulation of the relationship with the little moments and…there's not one big story, but there's lots of…little moments of, oh, this person's really obsessed with Disney*” (Clinician A75B688A).
Knowing someone beyond their diagnosis	“*the moments are going to happen because the doctor is not just singularly focused on, okay, let's see what your chart says. This is what it says. Here it is. Goodbye. No, he takes the time to say, Hey, what's going on? Did you play tennis or whatever? [B]y asking those questions, you're going to get some of those moments*” (Patient B8E0EF66).
	“*I think that I try, I can't always succeed, but I try to make time to talk to people, and talk less about their lisinopril that they were on 2 years ago, and more about what is really occupying their mind. And, I think that does create space at least for me. I'm very fortunate… to hear and to share in some of that*” (Clinician 57AC02C6).
	“*I think a doctor who has that extra skillset to say, [Patient Name], I love that dress today that you have on. Or remembers that one little tidbit, how are your kids doing? Or did he finally graduate college? Just one thing. It helps your healing… no matter how good the cancer part is, if our mind isn't, and we're not comfortable with our doctor, we can't heal. We can't get healthy*” (Patient 47677EE5).
Partnership, meeting patients where they are at	“…* having a conversation with somebody that I can feel, at least I've been able to talk with them about what I'm going through and what's happening to me, and they are talking back with me, you know, not belittling me or not putting me down*” (Patient 85C204C2).

## Theme 1: Sacred moment catalysts

Catalysts or common elements shared among these moments included pivotal points in clinical care (such as end of life), preparedness and expertise, organic moments, presence, shared characteristics, and continuity of care.

### Pivotal point in clinical care

The majority of participants identified sacred moments that took place in the context of a pivotal point in clinical care: initial cancer diagnosis, disease progression, end of life, or a good outcome related to cancer care. For example, one clinician shared an experience with a patient who had an excellent response to chemotherapy:

“*When I diagnosed her, it felt like it was like the end of the world. And then I saw her for her pre-op visit. She had neoadjuvant chemotherapy… and her tumor had shrunk. [S]he had had a giant tumor. And it disappeared. I went back to my note to make sure I knew what side her tumor had been on and went back to her, and it was gone. And just like in her eyes and the way that I felt of like, my God, like it might be gone…*” (Clinician-EEE8F431).

### Preparedness and expertise

Participants identified that conveying a sense of preparedness for a clinical visit such as knowledge of prior clinical events, and being able to present clinical expertise created space for sacred moments:

“*It always helps when somebody knows… your background and knows your latest numbers… when they come to you with that, it's like, all right, you‘re on my side and you know my history and you're just sticking with me*” (Patient-AF113363).

“*I think that is also something that instills a lot of trust in people when you come in to a visit educated. All that together lays the groundwork for some degree of trust that the patient already has in you… just based on your level of preparedness for the visit…*” (Clinician-C15EB1A4).

### Organic

Some participants highlight that sacred moments are spontaneous, without any identifiable drivers:

“*I don't know if it's something you actively seek out or it just kind of happens when it happens*” (Clinician-FD81F3A0).

### Being present

Participants described moments of presence and time spent in an encounter as foundational for experiencing a sacred moment:

“*Whenever we meet… it's my time. I never feel like the doctor is rushed. If we need to speak to him for an hour, we know he'd be there for an hour. I never had that rushed feeling that I have some other specialties where it's just… I gave you my 30 s, next, but he will take the time. And never, ever, ever have I ever felt rushed*” (Patient-B8E0EF66).

### Shared characteristics

Sacred moments emerged when clinicians and patients discovered shared experiences, allowing for genuine points of recognition and commonality in each other. Some shared that sacred moments took place when they were reminded of themselves:

“*I do think that it was because she was young. I think she was my age and she was a young woman. There was a relational thing like that*” (Clinician-A75B688A).

“*It gives you a better connection with the doctor, realizing that, he has the same type of thing going on in his life that we have going on in ours*” (Patient-0862035D).

### Continuity

Many participants discussed that longstanding relationships were a driver for experiencing sacred moments, noting the expanse of clinical moments that took place during that period:

“*[P]art of it's the longevity of the relationship. The fact that I had many interactions with her over the course of months and longer. And not just the frequency or longevity, but also the, the course of her illness*” (Clinician-FD81F3A0).

## Theme 2: Impacts of experiencing sacred moments

Participants identified various impacts of experiencing a sacred moment, some specific to clinicians including career fulfillment, some specific to patients including a sense of being cared for, and some shared impacts in both groups.

### Patient impacts: sense of being cared for

Patients responded that sacred moments allowed them to feel cared for as a whole person and be seen as a fellow human being:

“*I have felt that [my husband] is a human being first and a patient second. He is not cancer*” (Patient-B8E0EF66).

### Clinician impacts: career fulfillment

Common responses among clinicians included increased career fulfillment, meaning, and purpose:

“*This is why we do this… the ability to connect in a really powerful and meaningful way with an individual and families is deeply rewarding*” (Clinician-C96443BD).

Another clinician described that experiencing these moments of connection served as a counterbalance to more challenging moments and mitigated symptoms of moral injury and burnout experienced in a clinical career:

“*I think these are the moments that can carry me forward when I'm experiencing other less pleasant moments*” (Clinician-7D22F31F).

### Shared impacts: goal-aligned care

Both groups described that experiencing sacred moments allowed for a better understanding of their respective goals, which facilitated more aligned decision making to take place:

“*When you know who that person is as a person, and what matters most to them, I think you act differently, or you change behavior, or your decision making. I think it probably plays a large role into the actual decisions that are being made, and as such, the outcomes that matter to the patient*” (Clinician-510C0518).

### Shared impacts: trust building

When experiencing a sacred moment during clinical care both groups felt that trust was built, creating security in the relationship:

“*to have the sense that someone cares for you as an individual, not just as a clinical problem and listen to you and appreciates your humanity, I think gives people an enormous sense of relief and comfort and security. And again, trust*” (Clinician-C96443BD).

## Theme 3: Cultivation strategies

When asked about strategies to create space for sacred moments to occur, clinicians and patients identified the importance of building a relationship and knowing a patient beyond their medical diagnosis. Clinicians also highlighted the role of their training and workplace culture in valuing these exchanges.

### Culture and training

Many clinicians discussed the importance of their educators modeling and imparting the value of sacred moments, and because of this training culture, they have found it to be second nature to cultivate these moments in their own practice:

“…*having the people who train you also value these sorts of moments, but also just value the patient as the human being first in all of their care… because that has been emphasized to me so much throughout my training, that it's essentially just a part of how I provide care in general, as an automatic, as second nature*” (Clinician-192DE0E2).

One clinician hypothesized that as highly trained clinicians, creating structured trainings to codify and teach behaviors could increase the chances of experiencing sacred moments:

“…* it is good to look at this because maybe some of these things that are able to be sort of codified or defined better, can be taught*” (Clinician-640F317D).

### Building a relationship and knowing a patient beyond their diagnosis

Participants highlighted the importance of relationship building and time as factors that allow for sacred moments to occur, in addition to making the effort to know patients beyond their cancer diagnosis:

“*I think what creates relationships is one-on-one time, and your ability to speak with the patient, and spend time, and talk, not only about the numbers or the chemo, or the blood counts, but also share some human interaction. Learn about their interests, their goals, their family…*” (Clinician-1CB0F1D2).

One patient shared that experiencing these moments required investment from both parties to share of themselves, and found value in the stories that connected them to their clinician:

“*I think the best care providers look for ways to make connections. And it's a two way street. It's not just like us talking about us. … I keep track of how many iPads his kids have lost on transcontinental flights*” (Patient- B8E0EF66).

Another patient described the importance of an acknowledgment that they are more than only their cancer diagnosis:

“*I think a doctor who has that extra skillset to say… I love that dress today that you have on. Or remembers that one little tidbit, how are your kids doing?… Just one thing. It helps your healing…*” (Patient-47677EE5).

## Discussion

Our single-site mixed-methods study found that sacred moments are commonly experienced by clinicians and their patients in oncology, consistent with findings in internal medicine and radiation oncology (Quinn et al., 2023; Ameling et al., 2025; Saint et al., 2024). Our study identified several catalysts for sacred moments, revealing that profound connections often emerge from simple interactions. The salience of clinician presence and preparedness for clinical encounters highlights a potential therapeutic intervention of intentional engagement that may mitigate against time constraints commonly described in oncology care (Schenkel et al., 2025; Gajra et al., 2020). Notably, our findings suggest that these connections do not require time-intensive history taking or complex interventions, but rather brief acknowledgments of the person beyond the diagnosis and momentary bridges that honor shared human experiences. Powerful catalysts for meaningful connection included simple acts such as recalling details about a patient's hobby, sharing a personal anecdote about one's family, or complimenting a patient's clothes (Wolpaw and Shapiro, 2014; Safder, 2018; Saint et al., 2017). The ability to communicate clinical expertise while simultaneously engaging in these casual exchanges may create the perception of expanded time even within time-limited encounters. Our finding that sacred moments occur during pivotal clinical junctures emphasizes the heightened emotional significance of these transitions as crucial opportunities where authentic engagement can transform challenging conversations into meaningful connections.

Our findings suggest there are multifaceted impacts of sacred moments for both clinicians and patients, with emphasis on the reciprocal nature of genuine human exchange. The professional fulfillment clinicians derived from sacred moments suggests that these experiences may serve as a buffer to mitigate burnout, a significant concern in oncology where emotional demands are high and patients are often ill (Medisauskaite and Kamau, 2017; Blazin et al., 2021; Kleiner and Wallace, 2017). Fostering such moments may be associated with reduced burnout and increased job satisfaction (Rosa et al., 2022), career fulfillment (Vrontaras et al., 2025), and the integration of personal values with clinical practice (Anandarajah et al., 2025). These sources of purpose and resilience may help clinicians navigate the demands and challenges inherent to the practice of oncology. These findings align with meaning-centered frameworks of physician wellbeing, which propose that a sustained sense of purpose and connection in clinical work is protective against emotional exhaustion (Rosa et al., 2022; Shanafelt, 2009).

For patients, the cancer trajectory often triggers existential distress and a search for meaning, hope, and connection (Vehling and Kissane, 2018; Chen et al., 2022). We found that sacred moments fostered trust among patients, and affirmed their humanity beyond their cancer diagnosis or disease process. This trust may facilitate more effective communication about treatment options and goals of care. Creating better aligned clinical decision making, a cornerstone of patient-centered care, may enhance patient engagement, satisfaction, and trust in providers (Berman et al., 2016). The trust and sense of being cared for as a whole person that patients described is consistent with therapeutic alliance theory in which the relational bond itself drives benefit (Quinn et al., 2023; Sanft and Winer, 2023). Our findings suggest that sacred moments may function as key alliance-building experiences in oncology and may foster a more connected relationship between the clinician and patient (Quinn et al., 2023; Saint et al., 2024; Sanft and Winer, 2023).

These findings suggest that health systems should consider how organizational culture, training programs, and workflow design can preserve space for such deep moments of connection (Gilmartin and Saint, 2022). What might traditionally be dismissed as “small talk” may represent a therapeutically powerful intervention in cancer care (Wolpaw and Shapiro, 2014). Understanding patient values through approaches such as a validated dignity questionnaire (Hadler et al., 2022) may complement these organic moments of connection by providing clinicians with structured insights into patient priorities. This study adds to the evidence base regarding the importance of sacred moments by specifically characterizing sacred moments in oncology, where the burden of illness is profound and the clinician-patient relationship is often longitudinal. It is strengthened by the use of both quantitative and qualitative data and expansion of an established structured interview guide (Quinn et al., 2023). Future research should explore interventions to facilitate the occurrence of sacred moments and evaluate their long-term impact on both clinician and patient outcomes such as burnout, career longevity, patient satisfaction, and treatment adherence.

## Limitations

There are several limitations to our exploratory study. Our single-center design may limit generalizability and may not be reflective of patients or providers in other settings. The consistency of our themes with prior studies, however, suggests broader applicability. All clinicians in our cancer center were invited to participate voluntarily, and it is possible that those who have experienced sacred moments most frequently or who most value sacred moments were more likely to participate. As recruitment of clinicians was conducted through an open invitation by email and in-person departmental meetings, an exact response rate could not be calculated. Patients were recruited by a single investigator who is a clinician, which may have introduced selection bias and influenced the direction of conversation and positive associations with sacred moments during interviews. These potential influences were mitigated through regular discussion among the investigator team, independent coding by a second analyst who was not a clinician, and an iterative consensus based approach to theme development.

Participants were provided with a definition of sacred moments prior to interview to facilitate a shared understanding of the construct being studied, but this may have affected how participants interpreted and described their experiences, and may have contributed to the high reported prevalence of these moments. Our sample size was relatively small, though participants were recruited until we reached thematic saturation. Finally, the retrospective nature of all participant accounts may be subject to recall bias.

## Conclusion

Sacred moments are common and play an important role in oncology clinician career fulfillment and patient experience. Common elements that comprise these moments include preparedness, being fully present, and making small talk about life outside of the clinical setting. Deliberate adoption of these behaviors may lead to increased frequency of feeling connected between patients and their providers.

## Author's note

**Prior presentations**

(1) Presented as electronic poster at 2025 American Society of Clinical Oncology (ASCO) annual meeting: Yasin, F., Puklin, L., Quinn, M., Saint, S., and Sanft, T. B. (2025). Moments of connection between clinicians and patients in oncology: a mixed-methods exploratory study of sacred moments. *JCO* 43, e21004–e21004. doi: 10.1200/JCO.2025.43.16_suppl.e21004

(2) Presented as a poster presentation at the 10/2025 International Conference on Communication in Healthcare (ICCH): (a) Yasin, F., Puklin, L., Norman, K., and Sanft, T. “Moments of connection: a mixed-methods study of sacred moments between clinicians and patients in oncology,” in *International Conference on Communication in Healthcare 2025* (Ottawa, ON).

(3) Presented as a 45-min interactive workshop at the 9/2025 Gold Humanism Society (GHHS) Summit.

## Data Availability

The raw data supporting the conclusions of this article will be made available by the authors, without undue reservation.
