# Supplementary material for: Moments of deep connection between clinicians and patients in oncology: a mixed-methods study of sacred moments
Source: Front Psychol. 2026 Jun 9;17:1833697. doi: 10.3389/fpsyg.2026.1833697 (PMC13288769; doi:10.3389/fpsyg.2026.1833697)
Supplement: Supplementary file 1 [file Supplementary_file_1.pdf]

SUPPLEMENT:

Supplementary Table 1: Structured interview guide (adapted from Quinn et al.)

1. I will first define sacred or transcendent moments for us: These are moments of feeling connected with another person that are experienced while talking with a patient *[or provider in patient interviews]*, and are memorable, or "set-apart" from others. They can be shared or experienced independently (both parties don't have to agree that this is a transcendent moment). These moments can sometimes happen in times of great emotion – crisis, sadness, or great joy for example. Sometimes they are described as moments when "time stood still." Others describe them simply as moments of deep interconnectedness.
2. Have you ever experienced a sacred or transcendent moment in the healthcare setting? [If yes] Can you think of one that has happened recently or that stands out, and replay the moment as if you were telling me a story; think about the who, what, when, where. Be as specific as possible. If you need to write it down first, take a few moments to do that.
3. What was experiencing that moment like for you? How did it make you feel? What do you think makes it a moment "set-apart" from others?
4. How could you cultivate or increase the chances of experiencing these moments with your [patients/healthcare providers]?
5. What are the benefits from these moments?
6. Do you think these moments can impact overall clinical care? [if yes] In what ways do you think these moments impact overall clinical care?
7. What factors or conditions would need to be in place to increase the chances of experiencing these moments with your [patients/healthcare providers]?

Supplementary Table 2: Clinician Survey

1. **Age**: _______________

2. **Gender identity:**

Male	Female	Non-binary	Prefer not to answer

3. **Year in practice:**

Years post-training (select one):

0-5 years	5-10 years	10-15 years	>15 years

If still in training (select one):

PGY-4	PGY-5	PGY-6	≥ PGY-7

4. **Specialty**:

Hematology	Oncology	Hematology and Oncology	Hospice and palliative medicine

5. How often would you say you experience sacred moments in your clinical practice?

1	2	3	4	5
Never	Rarely (yearly or less)	Sometimes (monthly)	Often (weekly)	Always (once or twice per clinic)

1

6. When I experience a sacred moment, I feel professionally fulfilled.

1	2	3	4	5	6
No, strongly disagree	No, disagree	Neutral, neither agree or disagree	Yes, agree	Yes, strongly agree	Don't know

7. When I experience a sacred moment, I remember why I went into Hematology/ Oncology.

1	2	3	4	5	6
No, strongly disagree	No, disagree	Neutral, neither agree or disagree	Yes, agree	Yes, strongly agree	Don't know

8. When I experience a sacred moment, I feel connected to my patient.

1	2	3	4	5	6
No, strongly disagree	No, disagree	Neutral, neither agree or disagree	Yes, agree	Yes, strongly agree	Don’t know

9. My work is meaningful to me

1	2	3	4	5	6
No, strongly disagree	No, disagree	Neutral, neither agree or disagree	Yes, agree	Yes, strongly agree	Don’t know

10. I am contributing professionally in a way that I value[44]

1	2	3	4	5	6
No, strongly disagree	No, disagree	Neutral, neither agree or disagree	Yes, agree	Yes, strongly agree	Don’t know

Supplementary Table 3: Patient Survey

1. **Age**: ____________

2. **Gender identity:**

Male	Female	Non-binary	Prefer not to answer

3. **Diagnosis:**
   a. **Cancer type**: ______________
   b. **Stage**:

I	II	III	IV	I don't know

   c. **Year of diagnosis**: ______________

4. How often would you say you experience sacred moments with your clinical team?

1	2	3	4	5
Never	Rarely	Sometimes	Often	Always

5. When I experience a sacred moment, I feel connected to my oncology provider.

1	2	3	4	5	6
No, strongly disagree	No, disagree	Neutral, neither agree or disagree	Yes, agree	Yes, strongly agree	Don't know

6. I have trust in my oncology care team.

1	2	3	4	5	6
No, strongly disagree	No, disagree	Neutral, neither agree or disagree	Yes, agree	Yes, strongly agree	Don't know

7. I feel cared for by my oncology care team

1	2	3	4	5	6
No, strongly disagree	No, disagree	Neutral, neither agree or disagree	Yes, agree	Yes, strongly agree	Don’t know
